# Pancreatic Pseudocyst: Therapeutic Dilemma

**DOI:** 10.1155/2012/279476

**Published:** 2012-04-17

**Authors:** A. K. Khanna, Satyendra K. Tiwary, Puneet Kumar

**Affiliations:** Department of General Surgery, Institute of Medical Sciences, Banaras Hindu University, Varanasi 221005, India

## Abstract

Pancreatic pseudocyst develops in both acute and chronic pancreatitis. It is an entity likely to either remain asymptomatic or develop devastating complications. Despite being diagnosed easily, treatment exercise is still at crossroads whether in the form of internal or external drainage or endoscopic, laparoscopic, or open intervention with a good radiological guidance. The therapeutic dilemma whether to treat a patient with a pancreatic pseudocyst, as well as when and with what technique, is a difficult one. This paper is intended to get information about diagnostic and therapeutic exercises most appropriate for acute and chronic pancreatic pseudocyst.

## 1. Background

 The first description of pseudopancreatic cyst dates back almost two and half centuries to 1761 A.D. by Cannon et al. [1]. The management of cystic changes of the pancreas is an old problem. Eugene Opie, at the beginning of twentieth century, was the first to distinguish true pancreatic cysts, which are, by definition, lined by epithelium, from pseudocysts, which are surrounded by a wall composed of collagen and granulation tissue. More than two centuries after the first description, some clear consensus and guidelines were evolved in the Atlanta classification of 1993 [2]. The Atlanta classification consists of four distinct disease entities: acute fluid collections that develop early in the course of acute pancreatitis and do not yet have a cyst wall; acute pancreatic pseudocysts, which arise as sequelae of acute pancreatitis or trauma, and whose wall consists of granulation tissue and extracellular matrix; chronic pancreatic pseudocysts, which arise as sequelae of chronic pancreatitis and are likewise surrounded by a wall; pancreatic abscesses, which are intra-abdominal collections of pus immediately adjacent to the pancreas, without any large areas of necrosis. Acute fluid collections, pancreatic pseudocysts, and pancreatic abscesses can be distinguished from one another by the history, imaging studies of the wall of the abnormality and its contents, and, if necessary, a needle aspiration of the content [2].

## 2. Introduction

 Pseudocysts are formed after acute as well as chronic pancreatitis but more common after acute exacerbations of chronic pancreatitis than acute pancreatitis. There is lack of data containing randomized case-control studies, but numerous case series and reports indicate that pancreatic injury leads to pseudocyst formation. Pancreatic pseudocysts often arise as a complication of acute or chronic pancreatitis. The prevalence of pancreatic pseudocysts in acute pancreatitis has been reported to range from 6% to 18.5% [3, 4]. The prevalence of pancreatic pseudocysts in chronic pancreatitis range 20% to 40% [5]. Pancreatic pseudocysts most commonly arise in patients with alcoholic chronic pancreatitis (from 70% to 78%) [6]. The second most common cause is idiopathic chronic pancreatitis (from 6% to 16%), followed by biliary pancreatitis (from 6% to 8%). The incidence of pseudocyst is low ranging from 1.6 to 4.5% or 0.5 to 1 per 100000 adults per year [7, 8].

## 3. Classification

 D'Egidio and Schein, in 1991, described a classification of pancreatic pseudocyst based on the underlying etiology of pancreatitis (acute or chronic), the pancreatic ductal anatomy, and the presence of communication between the cyst and the pancreatic duct and defined three distinct types of pseudocysts [9]. TypeI, or acute “postnecrotic” pseudocysts that occur after an episode of acute pancreatitis and are associated with normal duct anatomy, rarely communicates with the pancreatic duct. Type II, also postnecrotic pseudocysts, which occurs after an episode of acute-on-chronic pancreatitis (the pancreatic duct is diseased but not strictured, and there is often a duct-pseudocyst communication). Type III, defined as “retention” pseudocysts, occurs with chronic pancreatitis and is uniformly associated with duct stricture and pseudocyst duct communication. 

Another classification, based entirely on pancreatic duct anatomy, is proposed by Nealon and Walser [10].

Type I: normal duct/no communication with the cyst.
Type II: normal duct with duct-cyst communication.
Type III: otherwise normal duct with stricture and no duct-cyst communication.
Type IV: otherwise normal duct with stricture and duct-cyst communication.
Type V: otherwise normal duct with complete cutoff.
Type VI: chronic pancreatitis and no duct-cyst communication.
Type VII: chronic pancreatitis with duct-cyst communication [10].

## 4. Diagnosis

 Pseudocyst of pancreas must be preceded by attacks of pancreatitis in either acute or chronic form. Most of the times, clinical, biochemical, and radiological evidence of pancreatitis present, but still a large number of patients may present with features of pancreatic pseudocyst without any documentary evidence of pancreatitis. One should always consider the possibility of a pseudocyst in a patient who has persistent abdominal pain, anorexia, or abdominal mass after a case of pancreatitis. Rarely, patients present with jaundice or sepsis from an infected pseudocyst [11]. In patients presenting with pancreatic cyst incidentally discovered on imaging, a crucial point is to define whether the patient has had prior history of pancreatitis [12]. Rarely, patients with large pancreatic pseudocyst may be asymptomatic occasionally. Tender abdomen with palpable mass is the positive finding on physical examination. Fever, icterus, and pleural effusion may be present in complicated pseudocyst. If pseudocyst ruptures then features of secondary peritonitis set in and presentation may be like septicaemic shock [11]. The diagnosis of a pancreatic pseudocyst is usually established by imaging studies, among which transabdominal ultrasonography is important as an initial investigation [13]. Computerized tomography (CT) is often the imaging method of choice, with 82% to 100% sensitivity and 98% specificity [14].

Biochemical parameters have limited role in diagnosis. Among remarkable parameters are serum amylase and serum lipase, which will be elevated in most cases. Liver functions are normally unchanged but may be deranged in cases where obstruction to the biliary tract occurs. Another thing to be considered is strong possibility of biliary peritonitis if liver parameters deranged. Other inflammatory marker C-reactive protein is raised and is of prognostic significance only. Elevated triglycerides and low serum calcium are indirect indictors of panreatic pseudocyst.

Differential diagnosis of pancreatic pseudocyst always may be of two possibilities either intrapancreatic lesions or extrapancreatic lesions.

Intrapancreatic diseases mimicking pancreatitis are:

1. pancreatitis (acute and chronic),
2. pancreatic necrosis,
3. pancreatic abscess,
4. adenocarcinoma of pancreas,
5. cystic neoplasm of pancreas,
6. pancreatic artery pseudoaneurysm.

Extrapancreatic diseases mimicking pancreatitis are

1. peptic ulcer disease,
2. acute cholecystitis and cholelithiasis,
3. gastric cancer,
4. abdominal aortic aneurysm,
5. ovarian cysts and carcinoma,
6. acute myocardial infarction,
7. pneumonia,
8. intestinal obstruction,
9. intestinal ischemia.

Among different imaging modalities, ultrasound (USG) is the foremost diagnostic tool and also useful pointer of diagnosis in most of the cases. It may be used in

- transabdominal USG,
- colour doppler study,
- duplex scanning,
- endoscopic USG.

Pancreatic pseudocyst appears as anechoic structure usually round or oval and surrounded by a smooth wall associated with distal acoustic enhancement on US examination. They are well defined and round or oval, and they are contained within a smooth wall. During the early phases of their development, pseudocysts can appear more complex, with varying degrees of internal echoes. If the earliest detection missed sometimes it may be due to excessive bowel gas. When necrotic debris or hemorrhage presents inside cyst or infection sets in then the interpretation on USG may be difficult. Color Doppler or duplex scanning should always be performed in cystic lesions to ensure that the lesion in question is not a giant pseudoaneurysm. Sensitivity rates for US in the detection of pancreatic pseudocysts are from 75% to 90%. Therefore, US is inferior to CT, which has a sensitivity rate of 90%–100%. US has several limitations, as compared with CT, in the initial diagnosis of a pseudocyst: the presence of overlying bowel gas decreases the sensitivity of US, and unlike CT, US examinations are highly operator dependent [15].

 A thick-walled, rounded, and fluid-filled mass adjacent to the pancreas on an abdominal CT scan in a patient with a history of acute or chronic pancreatitis is virtually pathognomonic for pancreatic pseudocyst. In acute manifestations when ileus or excessive gas shadow or bowel obstruction is a problem in USG evaluation, CT scan is definitely better and is purposeful in diagnosing pseudocyst. It is almost diagnostic and no other supplementary investigation that is required to confirm the diagnosis. Major advantage of CT scan is the detection of an objective and detailed anatomy as well as pathology. In addition to pancreas, extrapancreatic pathology as well as status of adjoining organs, for example, gallbladder, liver, common bile duct, stomach, and duodenum can be perfectly assessed. Contrast-enhanced CT is now the primary tool of investigation for initial diagnosis of pancreatic pseudocysts. USG should be done for the followup of asymptomatic pseudocysts or when diagnosis is uncertain. The only major limitation of CT scan is that it is unable to differentiate cystic neoplasm of pancreas from pseudocyst, and the main pathology to be missed is mucinous cystadenomas and intraductal papillary mucinous cystadenoma (IPMN) [16].

MRI and MRCP are accurate and sensitive diagnostic aids for defining the anatomy of duct better than any other diagnostic tool. But these are not used routinely as adequate information is obtained in maximum cases by CT, and very rarely ductal anatomy is needed to be calibrated with too much precision and MRI/MRCP is required. Pancreatic duct and biliary system are best visualized in detail although interpretation of integrity of pancreatic duct may be difficult [17]. MRCP techniques can also depict subtle branch-chain dilatation in chronic pancreatitis. MRI is also highly sensitive to the detection of bleeding with complex fluid collections.

 The role of endoscopic retrograde cholangiopancreatography (ERCP) is limited to some extent for therapeutic intervention rather than diagnostic purpose. It may help in planning an intervention after the increased use of endoscopic USG its role is gradually decreasing.

 Endoscopic ultrasound (EUS) is a test of choice to differentiate between cystic neoplasms of pancreas from pseudocyst. EUS is usually used as a secondary test to further evaluate pancreatic cyst detected by other imaging modality (US, CT or MRI). For the distinction of acute fluid collections from pancreatic abscesses and acute pancreatic pseudocysts, endosonography (EUS) has the highest sensitivity (93% to 100%) and specificity (92% to 98%). The diagnostic puncture of a pseudocyst under EUS guidance helps distinguish cystic malignancies from pseudocysts. A malignant lesion is more likely present when the carcinoembryogenic antigen (CEA) value exceeds 192 ng/mL and when the cyst contents are highly viscous [18].

Visualization of the pancreas via EUS provides high quality images due to the close proximity analysis, which are helpful to detect malignancy. An elevated CEA level on FNAC within the cyst fluid strongly suggests mucinous lesion [19, 20]. Amylase levels are usually high in pseudocysts and low in serous cystadenoma of the ultrasound transducer to the area of interest. Criteria suggestive of cystic neoplasm include a cyst wall thickness of greater than 3 mm, macroseptation (all cystic components more than 10 mm), the presence of a mass or nodule, and cystic dilation of the main pancreatic duct [19–21].

 Aspiration of cyst fluid under EUS guidance and biochemical analysis with molecular analysis helps in differentiating different cystic neoplasms of pancreas. Mutational changes and DNA content point towards malignancy.

## 5. Treatment

 Treatment of pseudopancreatic cyst comprises two aspects: supportive care or medical management and definitive care or surgical drainage.

 Intravenous fluids, analgesics, and antiemetics are the basic requirements. Low-far diet is given to patients who tolerate and intake. In patients with low or poor oral intake, support can be provided via nasoenteral feeding or total parenteral nutrition (TPN). To date, no studies have compared these two approaches in the seating of pancreatic pseudocyst, and the choice is based on availability and local preferences. If one can extrapolate from studies comparing the two modalities in the seating of acute necrotizing pancreatitis, one can expect that jejunal feeding will be related with fewer complications (infection) but may not be able to provide as much calories as TPN.

 The role of octreotide is still dubious as this has not been tested much with strong evidence in the literature. The rationale of using octreotide as a therapy for pancreatic pseudocyst is that it will decrease pancreatic secretions and aid in pseudocyst resolution. Unfortunately, this strategy has not been rigorously tested and only a handful of case series have been published [22, 23].

 Most pseudocysts resolve with supportive medical care. Vitas and Sarr followed over a period of 5 years 114 patients with the diagnosis of pancreatic pseudocyst [24]. Forty-six patients underwent primary operative therapy, with 13% undergoing emergency operations for pseudocyst-related complications. Morbidity occurred in 26% of patients (emergency operations, 67%; elective procedures, 10%) without any mortality. The remaining 68 patients were initially treated with a nonoperative expectant approach. Severe and life-threatening complications in this group (followup for a mean of 46 month) occurred in only six patients (9%); 19 patients eventually underwent elective operations directed at either the pseudocyst or other complications related to pancreatitis. Overall, in patients managed by a nonoperative approach, resolution of the pseudocyst occurred in 57% of the 24 patients with satisfactory radiographic followup, with 38% resolving more than 6 months after diagnosis. Although patients eventually undergoing operation tended to have larger pancreatic pseudocysts than the patients managed successfully nonoperatively (6.9 cm versus 4.9 cm), no serious complications occurred in seven patients with pancreatic pseudocysts greater than 10 cm who were treated expectantly [24].

Large-sized and long-standing cysts are not likely to respond on conservative treatment and more likely to have complications during the course of the disease. Morbidity and mortality are more commonly found in this group. These patients need surgical intervention and usually managed surgically. But some studies say that size and duration never matter, and actually these patients too have excellent surgical results and do well. There are two definite conclusions that the presence and the severity of symptoms and complications are determinants of prognosis and course in pancreatitis [25–27].

## 6. Drainage Procedures

 Most of the symptomatic and complicated pancreatic pseudocysts need intervention in any form during the course of the disease. Intervention options are either guided endoscopically, radiologically, laparoscopically, or open/direct. To date, no prospective controlled studies have compared directly percutaneous, surgical, and endoscopic drainage approaches. As a result, the management varies based on local expertise, but in general endoscopic drainage is becoming the preferred approach followed by laparoscopic approach.

 There is no consensus regarding methods of intervention in pancreatic pseudocyst although there is no controversy with conservative treatment. Minimal intervention with maximal conservative approach remains the most widely acceptable option of therapeutic intervention in pancreatic pseudocyst. Small sized asymptomatic cysts need no intervention at all. Asymptomatic large-sized cyst should be intervened after six weeks only and in the meantime is must be under close monitoring to detect the earliest symptoms or complications. Only in symptomatic cases or if any complication develops, intervention is required before six weeks. Cyst of any size should be intervened once it becomes symptomatic or if complications develop irrespective of duration, size, or site. So two things are important determinants the regarding plan of management: size when it is more than five cm and duration when it is more that six weeks. 

### 6.1. External Drainage

 External drainage can be achieved radiologically by using CT or US guidance. In this technique, a drainage pigtail catheter is placed percutaneously into the fluid cavity, and the fluid is drained. Three-dimensional ultrasonography has been reported useful for the guidance of catheters into cyst cavities and avoiding vessels. When the drainage output becomes minimal, the catheter is removed. Contrast injection into the cyst cavity will demonstrate the size of the remaining cavity, and this finding can be used to monitor the progress. This technique is successful in resolving pseudocysts, but it has a high risk of infections. This technique is definitely a failure if the catheter tends to block repeatedly. It tends to create significant discomfort to the patient. Furthermore, the catheter tends to clog and may require repositioning and exchange. The reported long-term success rate of pseudocyst resolution for US-guided pseudocyst drainage is around 50%. Unsuccessful drainages are usually caused by large ductal leaks or obstruction of the main pancreatic duct. Percutaneous catheter drainage is contraindicated in patients who are poorly compliant and cannot manage a catheter at home. It is also contraindicated in patients with strictures of the main pancreatic duct and in patients with cysts containing bloody or solid material [28–30].

### 6.2. Surgical Drainage

 In cases of failure of external percutaneous drainage radiologically, this approach is applied either by open method or by laparoscopy. It can be a good option for the patients who cannot tolerate endoscopic drainage. Stoma is created between the most dependent part of the cyst and the adjoining stomach, jejunum, or ileum to provide effective drainage.

 For surgical drainage, either lap or open method can be opted as both are effective for relief, but laparoscopic approach definitely carries low morbidity and mortality as compared to open techniques. Surgical drainage, which is increasingly done laparoscopically with a cholecystectomy if needed is the preferred mode then open approach.

 External drainage of pseudocyst should only be carried out in case of emergency relief of severe symptoms and sepsis. Otherwise, EUS or surgical drainage are the procedures of choice. Blind external drainage when duct status is unsure results in difficult-to-manage pancreatic fistulae [31].

### 6.3. Endoscopic Drainage

 Endoscopic drainage of pseudocysts is becoming the preferred therapeutic approach because it is less invasive than surgery. The intervention done is minimal and avoids the need for external drain and has a high long-term success rate. Internal drainage is accomplished with either a transpapillary approach with ERCP or direct drainage across the stomach or duodenal wall. A transpapillary approach is preferable when the pseudocyst communicates with the main pancreatic duct, usually in the gene of the pancreatic duct. This approach is also successful for patients with pancreatic duct disruption. The endoscopic approach is guided by the presence of a bulge into the lumen of the stomach or duodenum in order to determine the entry site for catheterization. This approach has several inherent risks, including missing the pseudocyst, injuring intervening vessels, and suboptimal placement of the drainage catheter [32]. Therapeutic echoendoscopes now make it possible to treat pseudocysts with EUS-guided transmural stenting [33]. Several series have described the deployment of a 7 Fr stent that is introduced with a needle-knife catheter [34]. A new large-channel echoendoscope allows the use of 10 Fr stent across the stomach or duodenum [35].

 In a large retrospective analysis of 603 patients who were undergoing EUS-FNA of pancreatic cysts, possible infection developed in only a single patient. The majority of patients in this series (90%) received antibiotic prophylaxis, most commonly a fluoroquinolone given for 3 days after the procedure, and this may possibly explain the low infection rate. The benefit of prophylactic antibiotics before an FNA of cystic lesions has not been evaluated by prospective randomized studies [36].

 The ASGE, in 2008, published the guidelines for prophylactic use of antibiotics for GI endoscopy. According to these guidelines, prophylaxis with an antibiotic, such as a fluoroquinolone, is administered before EUS-FNA of cystic lesions along the GI tract including pancreatic cyst. Antibiotics may be continued for 3–5 days after the procedure (supported by observational studies). The administration of antibiotic prophylax, a fluoroquinolone administered before the procedure and continued for 3 days after the procedure, is a reasonable regimen [37].

 Cahen et al. concluded that endoscopic drainage is an effective treatment for pancreatic pseudocysts and offers a definitive solution in almost three-quarters of the cases. The majority of the major complications might have been prevented by using pigtail stents instead of straight stents and by taking a more aggressive approach to the prevention and treatment of secondary cyst infection [38].

 Final decision on EUS versus surgical drainage is important and interesting as the decision making depends upon the profile of the patient. It is important to know that multiple procedures are sometimes necessary to ensure adequate drainage. Also when there is a large amount of solid debris, EUS drainage does not give good results. There has been significant technical advancement in EUS-guided drainage procedures with improved equipments and skill base. It is certain that EUS drainage will be more and more a preferred option over surgical drainage in the future too.

## 7. Complications

 Pancreatic pseudocyst needs close followup to early detect the most dreadful complications, which may be devastating if it remain unrecognized for long.

- *Infection*: infection occurs either spontaneously or after therapeutic or diagnostic manipulations. While infected pseudocyst can initially be treated with conservative means, a majority of patients will require intervention. Traditionally, surgery has been the preferred modality but endoscopic treatment is gaining acceptance. An external drainage may be necessary in selected situations such as when there is evidence of gross sepsis and the patient is too unstable to undergo surgical or endoscopic drainage [39].
- *Hemorrhage*: hemorrhage can greatly complicate the course of a pseudocyst and can be devastating [40]. The morbidity and mortality is very high because it can appear without warning and is usually due to erosion of a major vessel in the vicinity of the pseudocyst. If not recognized immediately, life of the patient may be jeopardized. Interventional radiology can play an invaluable role both in locating the source of bleeding and in embolisation of the bleeding vessel [41]. Without prior information of the bleeding point, surgical exploration can be hazardous and challenging.
- *Splenic infarction and thrombosis*: complications of pseudocyst include massive hemorrhage into the pseudocyst, sepsis with splenic infarction, and splenic vein thrombosis. The diagnosis of intrasplenic pseudocyst, based on clinical findings alone, is difficult to arrive at but should be suggested by the presence of a mass in the left upper quadrant. Sonography and computerized axial tomography may be particularly helpful in confirming splenic involvement. Selective celiac arteriography should be performed whenever splenic involvement is suggested in order to confirm the diagnosis and to search for pseudoaneurysm formation. Urgent surgical intervention is usually warranted in view of the high incidence of serious complications and the propensity toward rapid clinical deterioration. Resection of the pseudocyst by splenectomy and distal pancreatectomy is the treatment of choice [42].
- *Rupture*: rupture of a pseudocyst can have either a favorable or an unfavorable outcome, and this depends on whether it ruptures into the gastrointestinal tract, into the general peritoneal cavity, or into the vascular system. Rupture into the gastrointestinal tract either results in no symptoms or leads to melaena or hematemesis that usually requires urgent measures. Rupture into the general peritoneal cavity results in features of peritonitis and occasionally hemorrhagic shock. Emergent surgical exploration is usually required. While an internal drainage should always be aimed for, usually a thorough abdominal lavage and external drainage are all that can be achieved safely [43, 44].
- *Biliary complications*: biliary complications occur due to a large cyst in the pancreatic head region obstructing the common bile duct and resulting in obstructive jaundice. Therapeutic endoscopy with short-term biliary stenting is valuable in this situation. It can be retained until either the pseudocyst resolves or is treated by intervention [45, 46].
- *Portal hypertension*: portal hypertension can result from compression or obstruction of the splenic vein/portal vein either by the cyst alone or by the cyst in conjunction with underlying chronic pancreatitis. In this situation, surgery appears to be the only treatment modality available, and an appropriate surgical procedure can effectively treat this form of portal hypertension [47].
- *Gastric outlet obstruction*: pseudocysts around the head of the pancreas are likely to cause gastric outlet obstruction. Once the features of gastric outlet obstruction develop, it needs certainly intervention and decompression or drainage of the cyst.

## 8. Conclusion

 Pancreatic pseudocysts are the most common cystic lesions of the pancreas, accounting for 75%–80% of such lesions. The most common symptoms are abdominal pain, nausea, and vomiting, although they can be asymptomatic. Abdominal CT is an excellent choice for initial imaging. EUS plays an important role in differentiating pseudocyst from other cystic lesions of the pancreas and can greatly assist in transmural endoscopic drainage. Initial management consists of supportive care. Persistent symptoms and the development of complications warrant invasive intervention. The endoscopic and minimally invasive therapeutic procedures for the drainage of pancreatic pseudocysts are superior to open surgical techniques with respect to their success, morbidity, and mortality rates, but they cannot always be performed. In making treatment decisions, it is important to recall that 50% of pancreatic pseudocysts do not require any intervention and can be successfully managed by a wait-and-watch approach. Laparoscopic and endoscopic drainages have comparable success rates, while that of transcutaneous drainage are somewhat worse. Thus, the choice of technique depends very heavily on the experience of the treatment center. The surgical, percutaneous, and endoscopic pseudocyst drainage procedures have not been directly compared in high-quality prospective randomized studies and the preferred approach varies based on patient preferences and local expertise.
